# S100A11 sustains STRAP stability to drive PI3K/AKT/NF-κB feedforward signalling circuit activation in colorectal cancer progression

**DOI:** 10.1038/s41419-026-09117-x

**Published:** 2026-08-12

**Authors:** Jie Li, Han Yang, Ziyuan Xu, Hao Dong, Zhaojun Kou, Linlin Lu, Xuezhen Ma

**Affiliations:** 1 Qingdao Cancer Prevention and Treatment Research Institute, Qingdao Central Hospital, Qingdao University, University of Health and Rehabilitation Sciences, Qingdao, 266042 China; 2 Department of Urology, Southwest Hospital, Army Medical University (Third Military Medical University), Chongqing, China; 3 Department of Urology, The Affiliated Hospital of Qingdao University, Qingdao, 266001 China; 4 Qingdao Cancer Prevention and Treatment Research Institute, Qingdao Central Hospital, University of Health and Rehabilitation Sciences, Qingdao, 266042 China

**Keywords:** Tumour biomarkers, Colorectal cancer

## Abstract

Colorectal cancer (CRC) remains one of the most prevalent malignancies worldwide, with persistently low rates of early-stage diagnosis, poor overall prognosis, and a propensity for therapeutic resistance—challenges that underscore the urgent need for deeper mechanistic insight. By integrating single-cell transcriptomic data (GSE161277) with multi-omics datasets from TCGA and GEO repositories, alongside an independent clinical cohort, we identified S100A11 as a consistently overexpressed gene in CRC that correlates significantly with adverse patient outcomes. Functional studies established that S100A11 drives tumour progression by promoting cell proliferation, suppressing apoptosis, and inducing epithelial-to-mesenchymal transition (EMT). Mechanistically, S100A11 engages the WD6-7 domain (residues 221–302) of STRAP through direct physical interaction, thereby recruiting the deubiquitylase OTUB1 to shield lysine K246 of STRAP from K63-linked polyubiquitination. This protection stabilises STRAP and facilitates its association with PI3K, culminating in activation of the PI3K/AKT/NF-κB cascade and nuclear translocation of p65. We further demonstrate that p65 binds directly to the promoter regions of both S100A11 and STRAP, transcriptionally upregulating their expression and establishing a self-reinforcing feedforward loop that sustains pathway activation. Collectively, these findings delineate a hierarchical regulatory network centred on the S100A11/STRAP/NF-κB axis and offer a mechanistic framework with potential implications for CRC diagnosis and targeted intervention.

## Introduction

Colorectal cancer (CRC) ranks third in global cancer incidence and second in cancer-related mortality [1]. Despite notable progress in clinical diagnostics and multimodal therapy that has modestly improved patient survival, the overall prognosis for CRC remains poor. Dysregulated intracellular signalling is central to CRC pathogenesis [2]; sustained PI3K/AKT/NF-κB axis activity, in particular, has been linked to tumour cell proliferation, invasiveness, and chemotherapy resistance [3]. How this pathway becomes aberrantly activated upstream, however, is not yet fully understood. A clearer picture of the molecular events driving CRC initiation and progression is therefore needed—both to expose vulnerabilities amenable to therapeutic targeting and to refine our broader understanding of CRC regulatory networks.

The S100 protein family comprises low-molecular-weight calcium-binding proteins that influence cytoskeletal dynamics, cell-cycle progression, and inflammatory signalling across a range of malignancies [4]. Within CRC, several family members have been linked to disease progression: S100A4 promotes metastasis by facilitating EMT [5], while S100A8/S100A9 remodel the tumour-inflammatory microenvironment via RAGE receptor signalling [6]. S100A11 (also designated S100C or Calgizzarin) is a functionally important member of this subfamily involved in calcium signalling and the regulation of cell proliferation via multiple downstream effectors, with emerging recognition as a tumourigenesis marker across cancer types, and has been recognised as a potential marker of tumourigenesis and prognosis across several cancer types [4]. Cross-cancer mechanistic studies have begun to illuminate how S100A11 exerts these effects: in hepatocellular carcinoma, it accelerates tumour growth by reprogramming hepatic lipid metabolism and promotes EMT through the TGF-β pathway [7, 8]; in pancreatic ductal adenocarcinoma, it activates the pentose phosphate pathway to sustain malignant behaviour [9]; in gastric cancer, it drives progression by inducing EMT and blunting chemosensitivity [10]; and in prostate cancer, it fosters tumourigenicity partly by suppressing T-cell infiltration [11]. In CRC specifically, elevated S100A11 expression, detectable in both serum and tissue, has been reported to track with disease stage and to carry diagnostic value for assessing lymph node metastasis [12–14]. Despite this, the precise molecular circuitry through which S100A11 propels CRC progression has not been systematically delineated.

STRAP (serine-threonine kinase receptor-associated protein) is a WD40 repeat-domain scaffold protein whose expression is upregulated in CRC and transformed lung epithelium, where it contributes to proliferation and invasion through both TGF-β-dependent and -independent mechanisms [15]. How STRAP protein stability is regulated upstream, and how STRAP functionally connects to the PI3K/AKT pathway, remain open questions. OTUB1 is a deubiquitylase with well-documented oncogenic activity in multiple tumour types, largely achieved by antagonising proteasomal degradation of key substrates [16], yet its potential role in regulating STRAP stability in CRC has not previously been explored.

In the present study, analysis of single-cell transcriptomic data spanning multiple stages of colorectal neoplastic progression identified S100A11 as a candidate driver gene whose elevated expression correlated significantly with poor patient prognosis. Mechanistic dissection revealed that S100A11 binds directly to the 221–302 aa (WD6-7) domain of STRAP, recruiting the deubiquitylase OTUB1 to protect STRAP at lysine K246 from ubiquitin-mediated degradation. This stabilisation of STRAP in turn facilitates its interaction with PI3K, activating the PI3K/AKT/NF-κB cascade and driving CRC cell proliferation and EMT. We further found that NF-κB p65 transcriptionally upregulates both S100A11 and STRAP by occupying their respective promoter regions, thereby establishing a self-reinforcing feedforward loop that sustains pathway activity. Together, these findings delineate the hierarchical regulatory logic of the S100A11/STRAP/NF-κB axis, offer new perspective on the complexity of CRC signalling networks, and provide experimental support for targeting this axis as a therapeutic strategy. The data also raise the prospect that components of the S100A11/STRAP axis may serve as clinically useful biomarkers for CRC diagnosis and treatment stratification.

## Results

### S100A11 emerges as a key oncogenic determinant in colorectal carcinogenesis via single-cell transcriptomic analysis

We obtained single-cell RNA sequencing (scRNA-seq) data of normal colorectal tissues, colorectal adenomas, and colorectal carcinomas from the Gene Expression Omnibus (GEO) database (GSE161277). Data were visualised by nonlinear dimensionality reduction using the RunUMAP function, and cell clusters were delineated with FindAllMarkers, yielding thirteen distinct subclusters defined by characteristic gene expression signatures (Supplementary Fig. 1A–B). UMAP plots and bubble charts display the principal marker genes for each major cell subtype(Supplementary Fig. 1C-D). While most cell populations were observed across all three tissue types, their proportional abundances exhibited significant variations (Supplementary Fig. 1E).

Given the epithelial origin of CRC and to identify key genes driving colorectal malignancy, we subsequently focused our analysis on epithelial subpopulations. InferCNV analysis successfully distinguished benign from malignant epithelial cells within epithelial-originated populations (Supplementary Fig. 1F). Reclustering through dimensionality reduction partitioned epithelial cells into sixteen subclusters exhibiting heterogeneous malignancy scores. UMAP visualization demonstrated relative separation of normal, adenomatous, and carcinomatous tissues, with carcinoma samples containing residual benign epithelial cells and adenomas harbouring malignant-predisposed epithelial subpopulations (Supplementary Fig. 1G).

Subclusters C5, C11, and C15 displaying immunogenic signatures were excluded from further analysis due to potential technical artefacts (e.g., doublet cells, clustering errors) or biological heterogeneity. GSVA enrichment analysis of remaining subclusters revealed increased oncogenic pathway activation in high-malignancy-score subclusters (Supplementary Fig. 1G). Three characteristic subclusters were identified: C1 and C6 predominantly composed of adenoma components with divergent malignancy scores (C1: high malignancy; C6: low malignancy), and C4 representing high-malignancy carcinoma components (Supplementary Fig. 1H). These subclusters were selected for subsequent investigation.

To delineate molecular drivers of the adenoma-carcinoma sequence, we performed developmental trajectory analysis using the Monocle algorithm on subclusters C6 (low-malignancy adenoma), C1 (high-malignancy adenoma), and C4 (carcinoma). Low-malignancy C6 cells localised to the trajectory origin, whereas high-malignancy C1 and transitional C4 populations occupied opposing termini—a topology implying that divergent transcriptional programmes underpin malignant versus non-malignant cell fates (Supplementary Fig. 1I). We subsequently analyzed pseudotemporal changes in marker gene expression. Pseudotime ordering revealed bifurcated differentiation trajectories: a malignant transformation trajectory (Cell Fate 1) demonstrating predominant enrichment of oncogenic pathways, and a non-malignant trajectory (Cell Fate 2) (Supplementary Fig. 2A). Analysis of pseudotime-dependent gene expression patterns identified Cluster 2 genes exhibiting stage-specific differential expression and maximal enrichment in oncogenic pathways (e.g., MAPK, PI3K-Akt, ErbB). Among the top differentially expressed genes identified, S100A11 exhibited the most pronounced stage-stratified expression shifts, pointing to a potentially pivotal role in the malignant transformation of colorectal epithelium (Supplementary Fig. 2B).

### S100A11 is overexpressed in CRC and correlates with adverse prognosis

**Fig. 1:**
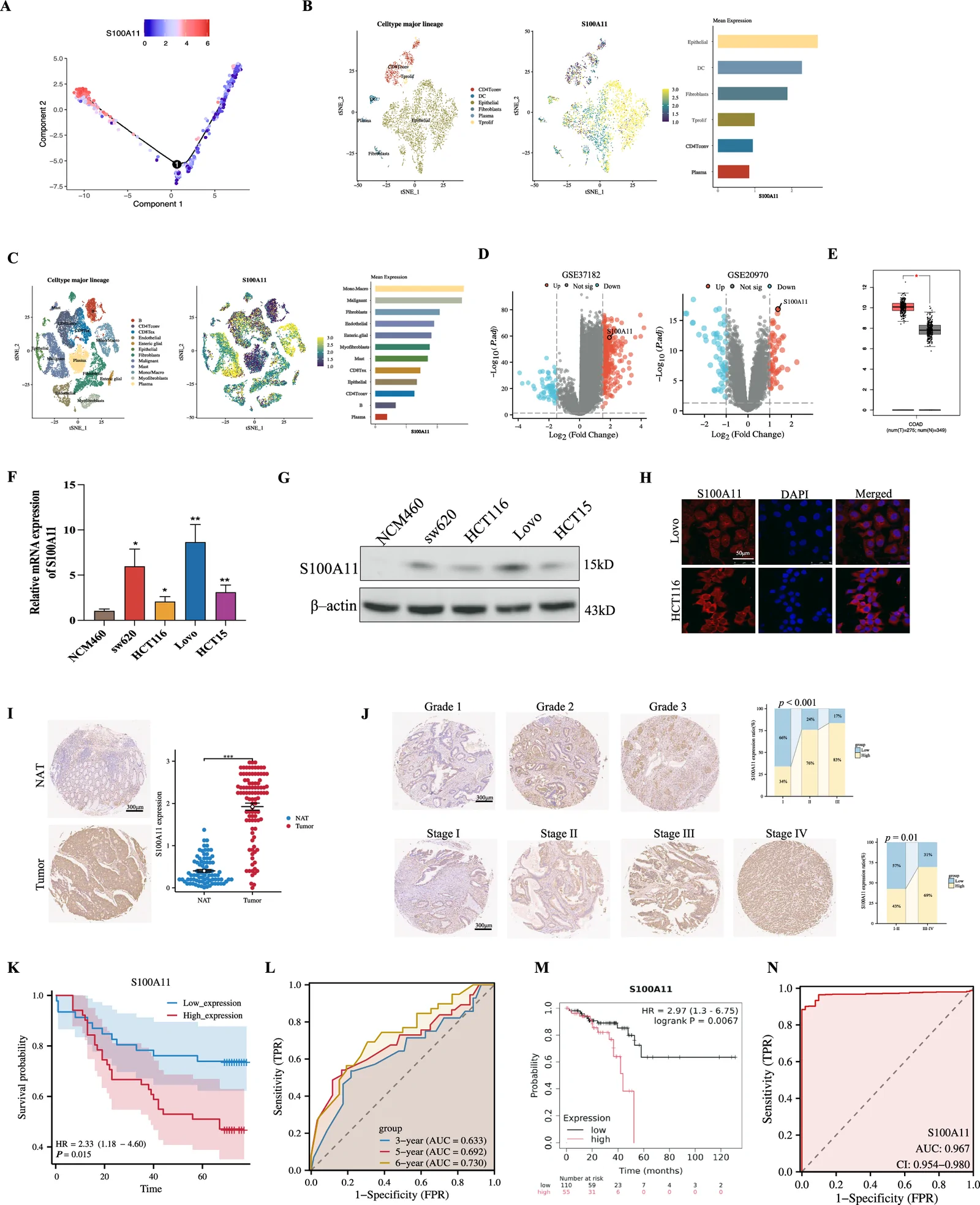
S100A11 overexpression correlates with adverse prognosis in colorectal cancer. **A** Pseudotemporal ordering of S100A11 expression during malignant transformation. Subcellular localization of S100A11 expression in scRNA-seq datasets GSE179784 (**B**) and E-MTAB8107 (**C**). **D** Volcano plot of DEGs in CRC transcriptomic datasets (GSE37182, GSE20907). **E** Differential S100A11 expression between tumour and adjacent normal tissues in TCGA-COAD. **F** RT-qPCR analysis of S100A11 mRNA levels in normal colon epithelial cells (NCM460) versus CRC cell lines. **G** Western blot validation of S100A11 protein expression. **H** Immunofluorescence imaging of S100A11 subcellular localization in Lovo and HCT116 cells (scale bar: 50 µm). **I** Representative IHC images (left) and quantitative analysis (right) of S100A11 expression in CRC versus normal tissues (scale bar: 300 µm). **J** Stage-stratified IHC analysis of S100A11 expression in CRC tissues (scale bar: 300 µm). **K** Kaplan-Meier survival curves showing prognostic significance of S100A11 overexpression. **L** ROC analysis evaluating S100A11’s diagnostic efficacy. **M** External validation of survival correlation using Kaplan-Meier Plotter. **N** ROC curve validation in TCGA-CRC cohort. *P < 0.05, **P < 0.01, ***P < 0.001.

Pseudotime analysis of gene expression revealed predominant enrichment of S100A11 in adenocarcinoma populations (Fig. 1A). Interrogation of CRC single-cell datasets (GSE179784, E-MTAB8107) demonstrated elevated S100A11 expression in epithelial-derived cells, particularly within malignant epithelial subpopulations (Fig. 1B, C). Differential expression analysis across GEO datasets (GSE37182, GSE20970) consistently identified S100A11 within upregulated gene clusters (Fig. 1D). TCGA CRC cohort analysis confirmed significant S100A11 overexpression in tumour tissues versus normal controls (Fig. 1E). Western blot and qRT-PCR validation in four CRC cell lines versus normal colonic epithelial cells (NCM460) revealed consistent S100A11 upregulation at both protein and mRNA levels (Fig. 1F, G). Immunofluorescence staining demonstrated that S100A11 exhibited primary cytoplasmic distribution accompanied by sporadic nuclear localization (Fig. 1H).

To evaluate clinical relevance, we performed immunohistochemical (IHC) staining on a tissue microarray (HColA180Su23) containing 97 matched CRC and adjacent normal samples (Supplementary Table 1). IHC quantification demonstrated significantly elevated S100A11 expression in CRC tissues compared to normal counterparts (Fig. 1I). Stage-stratified analysis revealed progressive S100A11 upregulation in advanced histopathological grades (grade 3) and TNM stages (stage IV), indicating a positive correlation with disease progression (Fig. 1J). Kaplan-Meier survival analysis demonstrated significantly reduced overall survival in patients with high S100A11 expression (Fig. 1K). Receiver operating characteristic (ROC) curve analysis highlighted S100A11’s prognostic value in predicting CRC survival outcomes (Fig. 1L). These findings were further corroborated in the TCGA CRC cohort (Fig. 1M, N).

To further investigate the clinical relevance of S100A11 expression in relation to CRC molecular subtypes, we analyzed its association with KRAS, NRAS, and BRAF mutation status and microsatellite instability (MSI) in the TCGA-COADREAD cohort. S100A11 mRNA expression did not differ significantly between KRAS-mutant and wild-type tumours, and no linear correlation was identified by Spearman analysis (Supplementary Fig. 3A–B). Likewise, no significant association was observed between S100A11 expression and NRAS mutation status (Supplementary Fig. 3C–D). These results indicate that S100A11 upregulation is independent of RAS mutational background, supporting its broad applicability as a prognostic biomarker and therapeutic target across distinct RAS mutation subtypes. Notably, S100A11 mRNA expression was significantly and inversely correlated with BRAF mRNA levels (Spearman R = −0.245, *P* < 0.001; Supplementary Fig. 3E–F), yet no significant difference was detected between BRAF-mutant and wild-type tumours, possibly reflecting the distinct MSI-H molecular context characteristic of BRAF V600E-mutant CRC, which warrants further investigation. Regarding MSI status, S100A11 expression showed a weak but significant inverse correlation with MSIsensor score (R = −0.09, *P* = 0.034; Supplementary Fig. 3G), indicating that tumours with higher instability burden tend to express modestly lower S100A11 levels. Furthermore, S100A11 was negatively correlated with MSH6 and PMS2 expression (both *P* < 0.05), but not with MLH1 or MSH2 (Supplementary Fig. 3H). Collectively, these data suggest that S100A11 high-expressing tumours predominantly occur in a microsatellite-stable, MMR-proficient molecular background.

### S100A11 promotes CRC proliferation and EMT features in vitro and in vivo

**Fig. 2:**
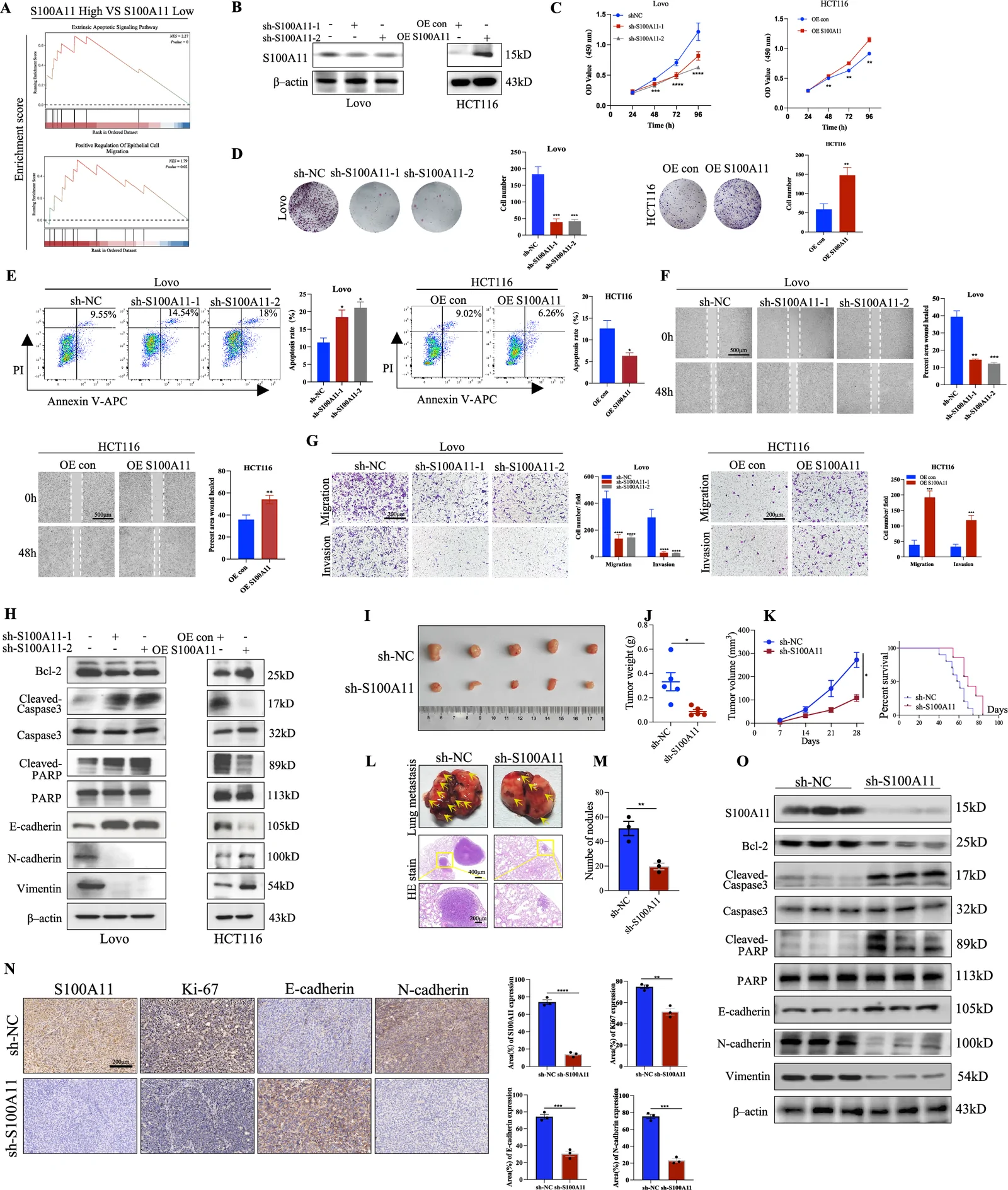
S100A11 promotes CRC cell proliferation and EMT in vitro and in vivo. **A** GSEA enrichment analysis of transcriptomic data from S100A11-overexpressing HCT116 cells versus controls. **B** Western blot validation of S100A11 knockdown in Lovo cells and overexpression in HCT116 cells. **C** CCK-8 proliferation assays in S100A11-knockdown Lovo and S100A11-overexpressing HCT116 cells. **D** Colony formation analysis post-S100A11 modulation. **E** Flow cytometry quantification of apoptosis rates. **F** Wound-healing assay assessing migratory capacity. **G** Transwell migration and invasion assays. **H** Western blot analysis of apoptosis- and EMT-related markers. **I** Representative images of subcutaneous xenografts in sh-NC versus sh-S100A11 Lovo groups. **J** Tumour weight quantification. **K** Tumour growth curves and mouse survival curves. **L** Lung metastasis specimens and H&E staining. **M** Metastatic nodule counts. **N** IHC analysis of S100A11, Ki-67, E-cadherin, and N-cadherin in xenografts (scale bar: 200 µm). **O** Western blot of proliferation and EMT markers in tumour tissues.

To investigate the biological role of S100A11 in colorectal cancer progression, we conducted high-throughput transcriptome sequencing on S100A11-overexpressing HCT116 CRC cells and control cells. GSEA enrichment analysis of the sequencing results revealed that S100A11 overexpression was enriched in apoptosis- and EMT-related phenotypes (Fig. 2A, Supplementary Fig. 4A). For Lovo cells with high endogenous S100A11 expression, specific shRNAs (sh-NC, sh-S100A11-1/2) were used to knock down S100A11. HCT116 cells with low S100A11 expression were infected with S100A11-overexpressing lentivirus. Transfection efficiency was validated at both mRNA and protein levels (Fig. 2B; Supplementary Fig. 4B).

CCK-8, colony formation, and flow cytometric apoptosis assays collectively showed that S100A11 knockdown suppressed Lovo cell proliferation and triggered apoptosis, while its overexpression produced the opposite phenotype (Fig. 2C–E). Wound-healing experiments revealed that S100A11 silencing markedly impaired scratch closure in HCT116 cells, whereas overexpression accelerated wound repair (Fig. 2F). Transwell assays corroborated these findings: S100A11 depletion reduced both migratory and invasive capacities in Lovo cells, while gain-of-function potentiated them (Fig. 2G). At the molecular level, qRT-PCR showed that knockdown downregulated Bcl-2, N-cadherin, and Vimentin while upregulating E-cadherin; overexpression yielded the inverse pattern (Supplementary Fig. 4C). Western blotting confirmed concordant changes in protein abundance, with knockdown additionally elevating the apoptotic markers Cleaved-PARP and Cleaved-Caspase-3 (Fig. 2H).

To assess the tumourigenic potential of S100A11 in vivo, we established subcutaneous xenograft models in BALB/c nude mice. Compared with controls, stable knockdown of S100A11 markedly suppressed tumour growth and reduced animal survival in Lovo cell-derived xenografts, whereas ectopic overexpression of S100A11 accelerated tumour proliferation in HCT116-derived grafts (Fig. 2I–L; Supplementary Fig. 4D). NCG mouse tail vein metastasis models demonstrated impaired lung metastasis capability with S100A11 knockdown and enhanced metastatic potential with overexpression (Fig. 2M, N; Supplementary Fig. 4E). IHC staining of subcutaneous tumours showed lower Ki-67 proliferation indices and higher E-cadherin levels in S100A11-knockdown groups, alongside elevated N-cadherin expression (Fig. 2O). Western blot results aligned with in vitro findings (Fig. 2P). Collectively, these results demonstrate that S100A11 promotes CRC cell proliferation and EMT characteristics.

### S100A11 interacts with STRAP to drive CRC proliferation and EMT

**Fig. 3:**
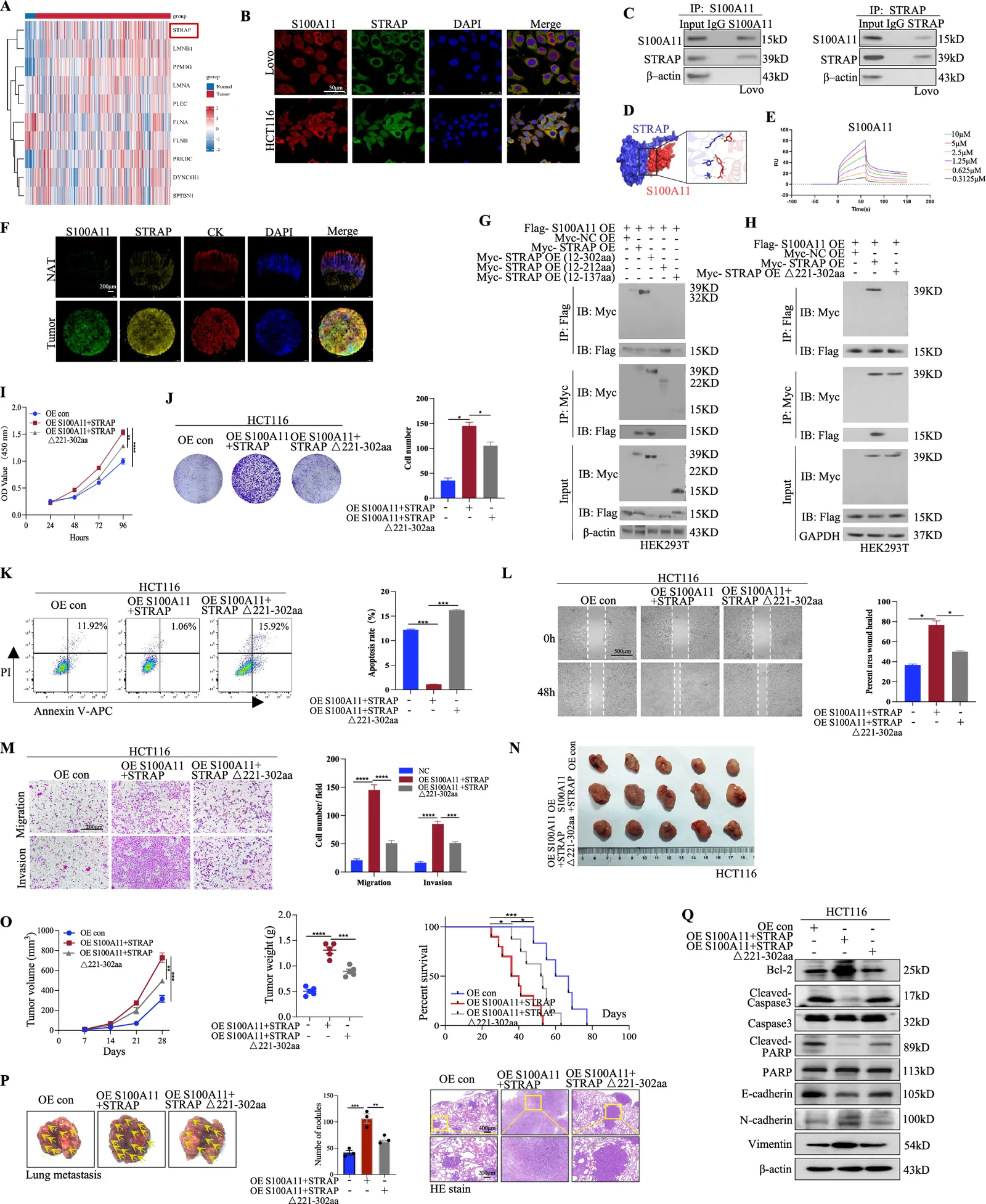
S100A11 interacts with STRAP to promote CRC cell proliferation and EMT. **A** Heatmap of top 10 S100A11-interacting proteins differentially expressed in TCGA CRC tissues. **B** Immunofluorescence co-localization of S100A11 and STRAP in CRC cells (scale bar: 50 µm). **C** Endogenous Co-IP and WB validation of S100A11-STRAP interaction in Lovo cells. **D** Molecular docking of S100A11-STRAP structural interfaces. **E** SPR analysis of direct binding between S100A11 and immobilised STRAP protein. S100A11 was applied as a twofold serial dilution series ranging from 0.3125 to 10 μM. RU, resonance units. **F** Multiplex immunofluorescence of S100A11, STRAP, and CK in CRC tissues (scale bar: 200 µm). **G** Co-IP analysis of the interaction between truncated STRAP constructs and full-length S100A11 in HEK293T cells. **H** Co-IP analysis of the interaction between STRAP mutants and full-length S100A11 in HEK293T cells. Proliferation and apoptosis of HCT116 cells in control, S100A11/STRAP co-transfection, and S100A11/STRAP Δ221–302 co-transfection groups, assessed by CCK-8 (**I**), colony formation assay (**J**), and flow cytometry (**K**). Migratory and invasive capacities of the three HCT116 groups evaluated by wound-healing assay (**L**) and transwell assay (**M**). Subcutaneous xenograft tumour images (**N**) and quantification of tumour weight, volume, and survival curves (**O**) for the three HCT116 groups in mice. **P** Lung metastasis specimens, HE staining, and statistics of lung metastasis counts in the three groups. **Q** WB analysis of proliferation and EMT markers in tumour tissues from the three groups.

To identify molecular partners underlying the pro-proliferative and metastatic effects of S100A11 in CRC, we established Flag-S100A11-overexpressing HCT116 cell lines and performed co-immunoprecipitation (Co-IP) coupled with mass spectrometry, identifying 734 candidate interacting proteins. The top 10 proliferation- and metastasis-associated proteins were selected based on identity scores (Supplementary Fig. 5A). Analysis of their expression in CRC versus normal tissues revealed STRAP as the top-ranked candidate (Fig. 3A). Immunofluorescence staining confirmed cytoplasmic co-localization of S100A11 and STRAP in CRC cells (Fig. 3B). Exogenous and endogenous Co-IP assays validated their interaction (Fig. 3C, Supplementary Fig. 5B), further supported by molecular docking of their structural interfaces (Fig. 3D). Direct binding between STRAP and S100A11 was further confirmed by surface plasmon resonance (SPR) (Fig. 3E).

Multiplex immunofluorescence analysis of clinical specimens (100 paired CRC and adjacent normal tissues; HColA160Su02) demonstrated a strong positive correlation between S100A11 and STRAP protein levels in CRC specimens (*P* < 0.001, R = 0.788; Supplementary Fig.4C). STRAP expression was elevated in advanced histopathological grades (grade 3) and TNM stages (III + IV) (Supplementary Fig. 5D), with high STRAP levels correlating with poor prognosis (Supplementary Fig. 5E). Tissue staining confirmed higher STRAP expression in CRC versus adjacent normal tissues, along with significant S100A11-STRAP co-localization in cancer regions (Fig. 3F). Notably, co-high expression of S100A11 and STRAP was predominantly observed in advanced histopathological grades (grade 3) and late-stage (III + IV) CRC tissues (Supplementary Fig. 5F). Patients with elevated co-expression exhibited significantly shorter overall survival compared to low-expression counterparts (Supplementary Fig. 5G). Stratified survival analysis based on S100A11/STRAP expression combinations (high/high, high/low, low/high, low/low) revealed the poorest prognosis in the dual-high subgroup (Supplementary Fig. 5H). Collectively, these data reveal a significant co-expression correlation between S100A11 and STRAP in CRC patient cohorts, suggesting their potential collaborative role in promoting oncogenic processes.

To delineate the structural basis of the S100A11-STRAP interaction, we generated serial STRAP truncation mutants (Supplementary Fig. 5I). Co-IP assays localized the binding interface to the 221-302aa region (WD6-7 domains) (Fig. 3G). Co-immunoprecipitation using the STRAP Δ221–302 deletion mutant further validated these findings: abolishing this interaction interface prevented co-precipitation of STRAP with S100A11 (Fig. 3H). Functionally, introduction of the Δ221–302 mutant significantly attenuated the gains in cell proliferation and EMT characteristics conferred by co-overexpression of S100A11 and wild-type STRAP (Fig. 3I–M). Conversely, in S100A11-depleted cells, re-expression of wild-type STRAP effectively restored proliferation and migratory capacity, whereas the Δ221–302 mutant failed to rescue either phenotype (Supplementary Fig. 6A–D). In vivo, tumours harbouring the Δ221–302 mutation exhibited reduced tumour volume, fewer pulmonary metastatic lesions, and extended survival compared with wild-type STRAP-expressing controls (Fig. 3N–P).

Consistent with in vitro findings, RT-qPCR and Western blot analyses of xenograft tissues demonstrated that the STRAP Δ221–302 aa mutation significantly suppressed the pro-proliferative and EMT features induced by S100A11 and STRAP overexpression (Fig. 3Q, Supplementary Fig. 5J). These data demonstrate that S100A11 promotes CRC proliferation and EMT through interaction with the STRAP 221–302 aa domain.

### S100A11 recruits deubiquitinase OTUB1 to regulate STRAP protein stability

**Fig. 4:**
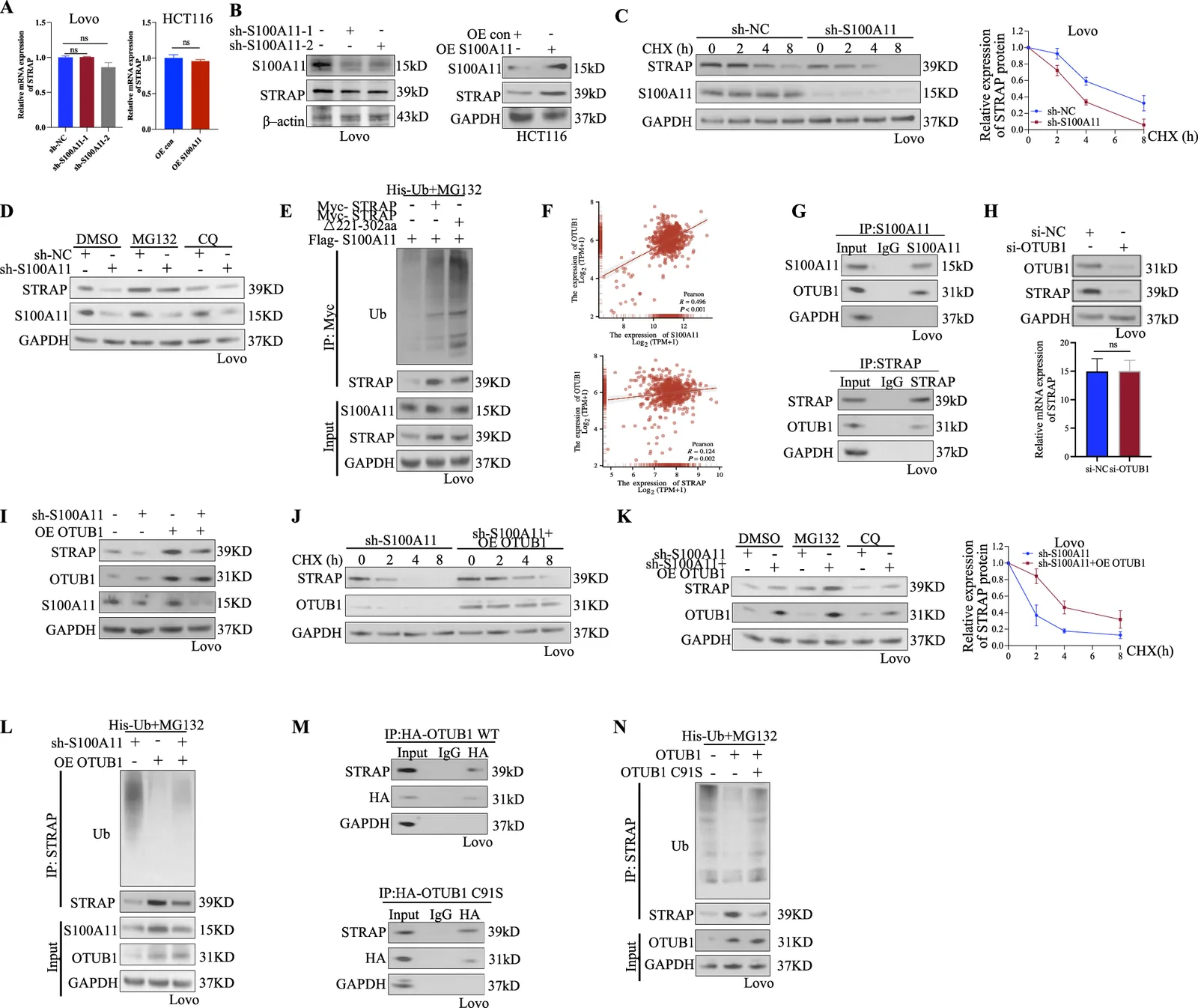
S100A11 stabilizes STRAP by recruiting OTUB1. **A** RT-qPCR analysis of STRAP mRNA levels upon S100A11 knockdown or overexpression. **B** WB validation of STRAP protein levels under S100A11 modulation. **C** Cycloheximide (CHX, 100 μg/ml) pulse-chase analysis of STRAP stability following S100A11 knockdown in Lovo cells (left). Representative quantification of STRAP expression levels (right). **D** WB analysis of STRAP stability in Lovo cells transfected with S100A11 shRNA or control, treated with DMSO, MG132 (10 µM), or chloroquine (CQ, 20 mM) for 6 h. **E** Ubiquitination levels of Myc-STRAP or Myc-STRAP Δ221-302aa in Lovo cells. All samples were pretreated with MG132 (10 μM) for 6 h. **F** Correlation analysis of OTUB1 with S100A11 and STRAP expression in TCGA-CRC data. **G** Co-IP and WB detection of endogenous interactions among OTUB1, S100A11, and STRAP in Lovo cells. **H** Effects of OTUB1 knockdown on STRAP mRNA and protein levels in Lovo cells. **I** WB analysis of STRAP expression in S100A11-knockdown Lovo cells with or without OTUB1 overexpression. **J** CHX pulse-chase analysis of STRAP stability in S100A11-knockdown Lovo cells overexpressing OTUB1 (left). Quantification of STRAP levels (right). **K** STRAP stability in S100A11-knockdown Lovo cells with OTUB1 overexpression, treated with DMSO, MG132 (10 µM), or CQ (20 mM) for 6 h. **L** Ubiquitination levels of STRAP in S100A11-knockdown Lovo cells with or without OTUB1 overexpression. All samples were pretreated with MG132 (10 μM) for 6 h. **M** Co-IP and WB analysis of interactions between STRAP and OTUB1 or OTUB1 C91S in Lovo cells. **N** Ubiquitination levels of STRAP in Lovo cells transfected with OTUB1 or catalytically inactive OTUB1 C91S. All samples were pretreated with MG132 (10 μM) for 6 h.

Next, we investigated whether S100A11 affects STRAP expression and stability in CRC cells. Knockdown of S100A11 significantly reduced STRAP protein levels, whereas overexpression of S100A11 increased STRAP protein abundance. Intriguingly, STRAP mRNA levels remained unchanged (Fig. 4A, B). These findings suggested that S100A11 modulates STRAP protein stability. Treatment of CRC cells with the protein synthesis inhibitor cycloheximide (CHX) revealed that S100A11 knockdown shortened the half-life of STRAP (Fig. 4C). As previously established, cellular protein degradation primarily occurs via the ubiquitin-proteasome system (UPS) or autophagy-lysosomal pathway [17].To determine the degradation pathway, we applied the proteasome inhibitor MG132 and the lysosomal inhibitor chloroquine (CQ). MG132, but not CQ, blocked STRAP degradation (Fig. 4D). Overexpression of S100A11 with a mutated STRAP (221-302aa) increased STRAP ubiquitination (Fig. 4E), indicating that S100A11 suppresses STRAP degradation via the ubiquitin-proteasome pathway rather than the autophagy-lysosomal route.

To define the mechanism by which S100A11 shields STRAP from ubiquitination, we focused on the interaction interface mapped to the WD6-7 domain (residues 221–302). Bioinformatic analysis across species identified lysine K246 as an evolutionarily conserved candidate site, and we generated a STRAP K246R point mutant alongside a K231R mutant as an intra-domain control. In S100A11-depleted Lovo cells, the K246R mutant displayed markedly reduced ubiquitination relative to wild-type STRAP (Supplementary Fig. 6E), implicating K246 as the principal ubiquitination site under S100A11 regulation. To determine the linkage topology of the modification, we employed ubiquitin mutants permissive for either K48- or K63-linked chain assembly. Abrogation of K63-linked ubiquitin (HA-Ub-K63) substantially diminished STRAP ubiquitination signal (Supplementary Fig. 6F), indicating that the modification is predominantly K63-linked and thus more likely to regulate STRAP activity and protein interactions than to target it for proteasomal degradation. At the functional level, rescue with the K246R mutant more effectively reversed the proliferative and migratory deficits caused by S100A11 depletion than rescue with wild-type STRAP (Supplementary Fig. 6A–D), providing functional corroboration that K246 is the dominant modification site. Taken together, these data indicate that S100A11, through its engagement with the WD6-7 domain, selectively protects STRAP at K246 from K63-linked polyubiquitination, thereby preserving STRAP protein stability.

To explore the underlying mechanism, we hypothesized that S100A11 recruits deubiquitinases (DUBs) to counteract STRAP ubiquitination, as S100A11 lacks intrinsic DUB activity. Mass spectrometry data identified OTUB1, a DUB of the OTU superfamily, as a candidate. Correlation analysis in TCGA_CRC samples showed positive associations among OTUB1, S100A11, and STRAP (Fig. 4F). Endogenous CO-IP confirmed interactions between OTUB1 and both S100A11/STRAP (Fig. 4G). OTUB1 depletion reduced STRAP protein abundance without affecting mRNA levels (Fig. 4H), while OTUB1 overexpression rescued the STRAP loss caused by S100A11 knockdown (Fig. 4I). CHX chase and MG132 treatment demonstrated that OTUB1 overexpression extended STRAP half-life and promoted protein accumulation; chloroquine (CQ) had no effect, pointing to proteasomal rather than lysosomal degradation (Fig. 4J–K). S100A11 knockdown enhanced STRAP ubiquitination, an effect reversed by OTUB1 co-expression (Fig. 4L). To confirm catalytic dependency, we employed the OTUB1 C91S mutant, in which substitution of the catalytic cysteine abolishes deubiquitylase activity [18]. OTUB1 C91S retained its physical association with STRAP (Fig. 4M) yet failed to stabilise STRAP protein levels compared with wild-type OTUB1 (Fig. 4N), establishing that the protective effect requires OTUB1 enzymatic function. Collectively, these results demonstrate that S100A11 stabilizes STRAP through recruitment of OTUB1, with this stabilization dependent on OTUB1 deubiquitinase activity.

### S100A11 drives CRC proliferation and EMT through PI3K/AKT/NF-κB pathway activation

**Fig. 5:**
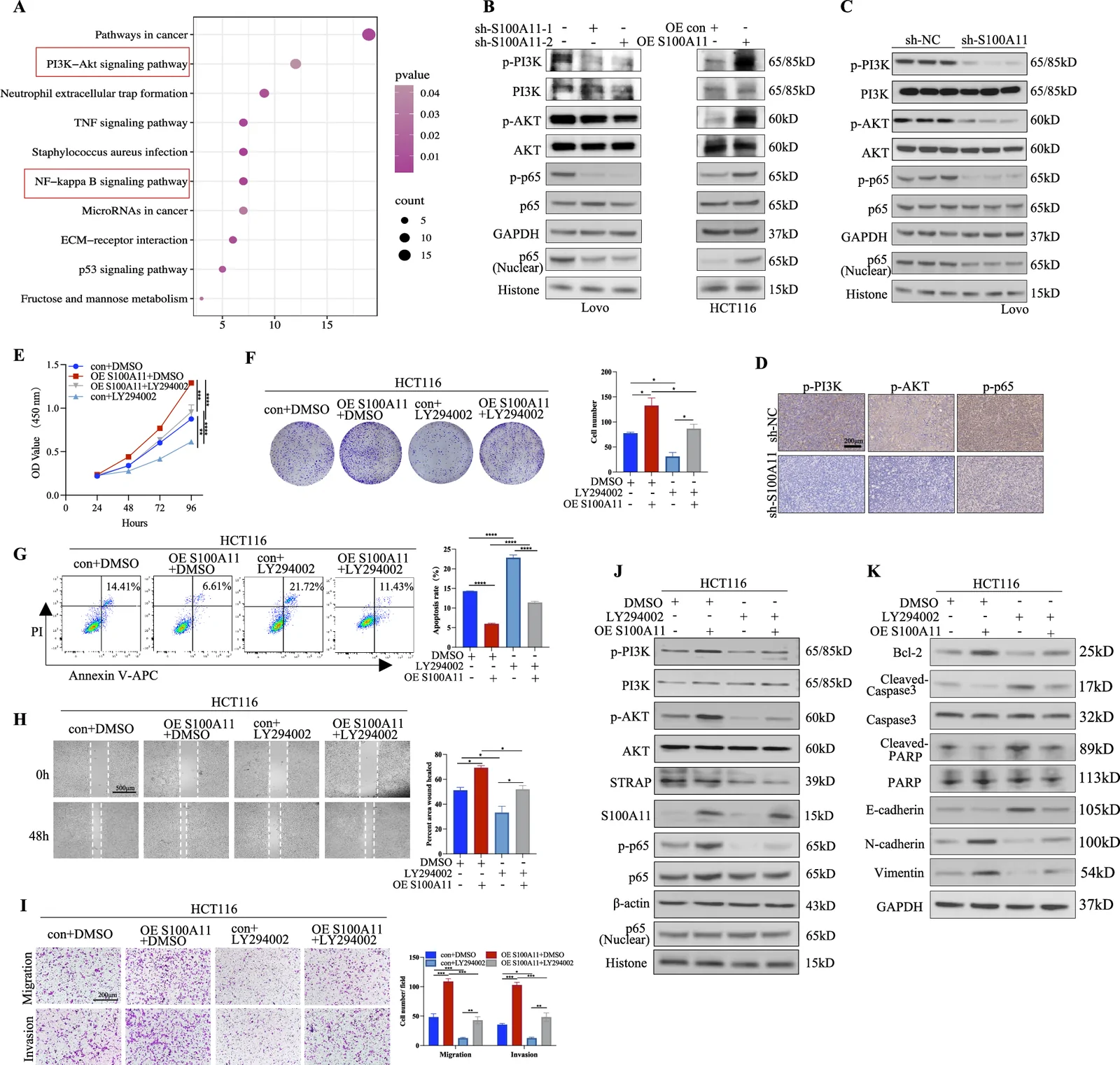
S100A11 promotes CRC proliferation and EMT by activating the PI3K/AKT/NF-κB signalling pathway. **A** KEGG enrichment analysis of high-throughput transcriptome sequencing data from S100A11-overexpressing HCT116 cells and control cells. **B** Western blot analysis of key proteins in the PI3K/AKT/NF-κB p65 signalling pathway following S100A11 knockdown or overexpression. **C** Western blotting of key PI3K/AKT/NF-κB p65 pathway proteins in tumour tissues from mice with S100A11 knockdown. **D** Representative IHC staining of phosphorylated PI3K/AKT/NF-κB p65 proteins in mouse tumour tissues. CCK-8 assay (**E**) and colony formation assay (**F**) demonstrating the effects of S100A11 overexpression (with or without 20 μM LY294002 inhibitor treatment) on HCT116 cell proliferation. **G** Flow cytometry analysis of apoptosis in HCT116 cells under the same experimental conditions. **H** Wound-healing assay evaluating migration capacity of HCT116 cells across experimental groups. **I** Transwell assay assessing migration and invasion abilities of HCT116 cells. Western blotting of key PI3K/AKT/NF-κB p65 pathway proteins (**J**) and apoptosis/EMT-related markers (**K**) in HCT116 cells under corresponding treatments. Data are presented as mean ± SEM; *P < 0.05, **P < 0.01, ***P < 0.001, ****P < 0.0001.

To further elucidate the oncogenic mechanisms of S100A11 in colorectal cancer, we performed KEGG pathway enrichment analysis on the aforementioned transcriptomic sequencing data. These data revealed significant enrichment of S100A11-regulated transcripts in PI3K/AKT and NF-κB gene sets (Fig. 5A). Given that NF-κB functions as a well-established downstream effector of PI3K/AKT—a cascade with critical roles in CRC proliferation and metastasis [19], we next examined the phosphorylation status of key pathway nodes. S100A11 knockdown reduced p-PI3K, p-AKT, and p-p65 levels without affecting the corresponding total proteins. Additionally, nuclear p65 expression decreased upon S100A11 silencing, whereas overexpression yielded opposite effects (Fig. 5B). These findings suggest S100A11 promotes NF-κB nuclear translocation via PI3K/AKT/NF-κB pathway activation, thereby enhancing CRC proliferative and metastatic capacities. Western blot and IHC analyses of xenograft tissues corroborated these trends (Fig. 5C, D). LY294002, a broad-spectrum PI3K inhibitor that blocks downstream pathway activity, was applied to S100A11-overexpressing cells. This treatment significantly suppressed CRC proliferative phenotypes in CCK-8 assays (*p* < 0.01), colony formation (*p* < 0.05), and flow cytometric apoptosis analysis (*p* < 0.01) (Fig. 5E–G), while markedly inhibiting EMT phenotypes in wound-healing (p < 0.01) and Transwell assays (*p* < 0.05) (Fig. 5H–I). Western blot analysis further demonstrated that LY294002 effectively abolished S100A11-overexpression-induced activation of the PI3K/AKT/NF-κB pathway and p65 nuclear translocation (Fig. 5J). Consistent with these findings, LY294002 neutralized the regulatory effects of S100A11 overexpression on proliferation- and EMT-related markers (Fig. 5K). These results collectively indicate that S100A11 maintains CRC proliferative and EMT phenotypes through PI3K/AKT/NF-κB signalling dependency.

### S100A11 facilitates STRAP-PI3K interaction to augment PI3K/AKT/NF-κB signalling activation

**Fig. 6:**
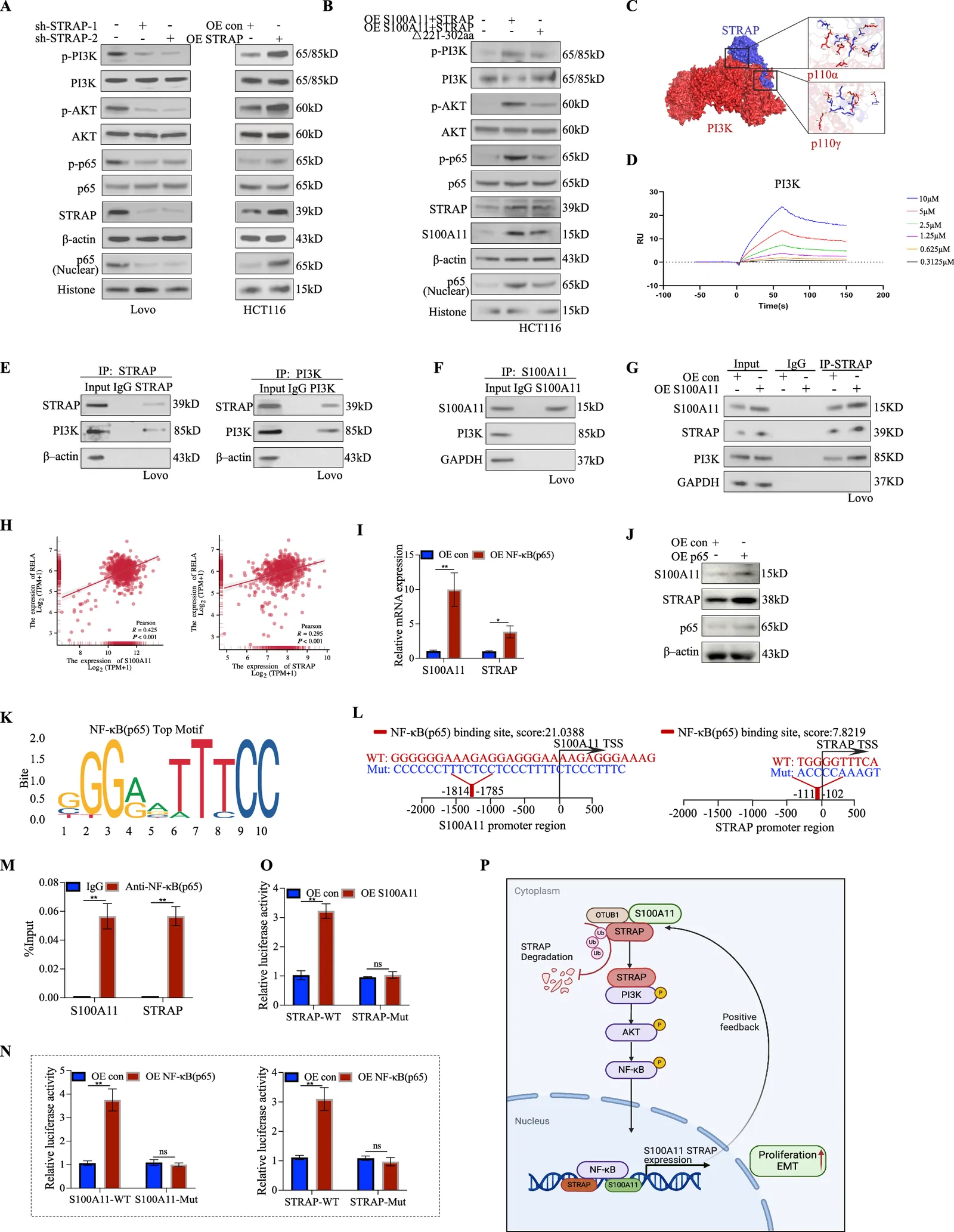
NF-κB p65 transcriptionally upregulates S100A11 and STRAP. **A** Western blot analysis of PI3K/AKT/NF-κB p65 pathway proteins following STRAP knockdown or overexpression. **B** Effects of STRAP or STRAP ∆221–302 overexpression on PI3K/AKT/NF-κB p65 pathway proteins in S100A11-overexpressing HCT116 cells. **C** Molecular docking of STRAP and PI3K three-dimensional structures. **D** SPR detection of direct binding of PI3K to immobilized STRAP. PI3K concentration range 0.3125–10 μM (twofold serial dilution), RU: resonance unit. **E** Co-IP and Western blotting validating endogenous interaction between STRAP and PI3K in Lovo cells. **F** Co-IP and Western blotting confirming endogenous interaction between S100A11 and PI3K in Lovo cells. **G** Co-IP and Western blotting showing STRAP-PI3K interaction in S100A11-overexpressing vs. control Lovo cells. **H** Correlation between NF-κB p65 and S100A11/STRAP expression in the TCGA_CRC dataset. RT-qPCR (**I**) and Western blotting (**J**) of S100A11 and STRAP mRNA/protein levels in NF-κB p65-overexpressing Lovo cells. **K** Predicted NF-κB p65 DNA-binding consensus sequences from JASPAR. **L** Top predicted NF-κB p65 binding sites within S100A11 and STRAP promoters. **M** ChIP-qPCR demonstrating direct binding of NF-κB p65 to specific loci in S100A11 and STRAP promoters in Lovo cells. **N** Dual-luciferase reporter assay showed the dual-luciferase activities after co-transfection of NF-κB p65 with the wild-type (WT) and mutant (Mut) 3’-UTRs of S100A11 and STRAP, respectively in HEK293T cells. **O** Dual-luciferase activity in S100A11-overexpressing vs. control HEK293T cells transfected with STRAP 3’-UTR WT or Mut. **P** Schematic illustrating S100A11-mediated molecular mechanisms driving CRC progression.

STRAP expression is upregulated in transformed epithelia of human colorectal and lung cancers, where it regulates cell proliferation and metastasis through both TGF-β-dependent and -independent mechanisms [20]. While the PI3K/AKT pathway represents a critical TGF-β-independent branch, the specific role of STRAP in this signalling axis remains less characterized compared to its TGF-β-related functions. Phosphorylation analysis of pathway nodes demonstrated STRAP-mediated activation of PI3K/AKT/NF-κB signalling and p65 nuclear translocation (Fig. 6A). Co-overexpression of S100A11 and STRAP synergistically enhanced PI3K/AKT/NF-κB phosphorylation and p65 nuclear accumulation, whereas mutation of the STRAP 221–302 aa domain significantly suppressed these effects (Fig. 6B).

To investigate direct regulatory relationships between STRAP and the PI3K/AKT/NF-κB pathway, protein structure-based molecular docking revealed physical interaction between STRAP and PI3K (Fig. 6C). SPR experiments confirmed the direct binding of STRAP to PI3K (Fig. 6D). Endogenous Co-IP assays confirmed STRAP-PI3K interaction but showed no direct binding between S100A11 and PI3K (Fig. 6E, F). Notably, S100A11 overexpression potentiated the STRAP-PI3K interaction (Fig. 6G).

### NF-κB p65 transcriptionally activates S100A11 and STRAP in colorectal cancer

NF-κB p65 (RELA), a pivotal transcription factor regulating proliferation- and metastasis-associated genes in cancer [21]. TCGA_CRC data demonstrate that S100A11 expression is positively correlated with NF-κB p65 (RELA) expressions (Fig. 6H). NF-κB p65 overexpression upregulated both mRNA and protein levels of S100A11 and STRAP (Fig. 6I-J), implying that NF-κB may exert transcriptional regulation over S100A11 and STRAP expression. JASPAR analysis revealed frequency matrices of NF-κB p65 binding motifs (Fig. 6K), with the highest-ranked predicted binding sites identified at positions –1785 to –1814 (sequence: GGGGGGAAAGAGGAGGGAAAAGAGGGAAAG) upstream of the S100A11 transcriptional start site and positions -102 to -111 (sequence: TGGGGTTTCA) upstream of the STRAP initiation site (Fig. 6L). Chromatin immunoprecipitation (ChIP) assays confirmed NF-κB p65 binding to both promoters (Fig. 6M). To functionally validate this transcriptional regulation, we generated luciferase reporter vectors harbouring intact (WT) or disrupted (Mut) NF-κB p65 binding motifs within the S100A11 and STRAP promoters. Dual-luciferase assays revealed that NF-κB p65 overexpression significantly enhanced luciferase activity driven by S100A11-WT and STRAP-WT promoters, while mutation of these motifs (Mut) abrogated responsiveness (Fig. 6N). Intriguingly, S100A11 overexpression amplified NF-κB p65-mediated transcriptional activation of STRAP (Fig. 6O). These findings delineate a self-reinforcing loop wherein NF-κB p65 directly binds to and transcriptionally activates S100A11/STRAP expression, establishing a positive feedback circuit in CRC pathogenesis.

To determine whether the pro-tumourigenic effects of p65 are mediated through its transcriptional targets S100A11 and STRAP, we re-introduced either protein individually into p65-depleted Lovo cells. p65 knockdown substantially impaired cell proliferation, clonogenic capacity, and invasiveness, and each of these defects was rescued by overexpression of either S100A11 or STRAP alone (Supplementary Fig. 6G–I). At the molecular level, the reductions in AKT phosphorylation and in the expression of Bcl-2 and E-cadherin observed upon p65 loss were similarly restored following add-back of either protein (Supplementary Fig. 6J). These results establish S100A11 and STRAP as indispensable downstream effectors of p65-driven CRC malignancy, and functionally validate the necessity of this feedforward loop. Extending this epistasis analysis, we found that direct expression of constitutively active myr-AKT or CA-p65 in S100A11-stable-knockdown Lovo cells was sufficient to reinstate the suppressed phenotypes (Supplementary Fig. 6A–D).

Collectively, the data support the following model: S100A11 binds and stabilises STRAP to activate PI3K/AKT; activated AKT drives nuclear translocation of NF-κB p65; p65 in turn transcriptionally upregulates both S100A11 and STRAP, completing a self-amplifying feedforward circuit that sustains CRC progression (Fig. 6P).

## Discussion

The molecular heterogeneity of CRC fundamentally limits the efficacy of single-target intervention strategies [22]—patients with distinct molecular subtypes frequently display divergent responses to the same pathway inhibitor. Starting from multi-stage CRC single-cell transcriptomic data and integrating TCGA/GEO multi-omics analyses with clinical cohort validation, the present study systematically delineates the mechanistic path by which S100A11 stabilises STRAP, facilitates STRAP–PI3K complex assembly, activates the PI3K/AKT/NF-κB cascade, and ultimately drives CRC cell proliferation and EMT. NF-κB p65 transcriptionally upregulates both S100A11 and STRAP by occupying their respective promoter regions, and the resulting feedforward loop renders pathway activation self-sustaining—a property that biologically accounts for the tight association between high S100A11 expression and persistently poor prognosis in CRC patients. Collectively, these findings uncover a previously unrecognised role and mechanistic basis for the S100A11/STRAP/NF-κB p65 feedforward circuit in CRC initiation and progression.

The S100 family encompasses an expansive subfamily of structurally related yet functionally divergent low-molecular-weight calcium-binding proteins (9–14 kDa). Through binding to target proteins and modulating intracellular signalling, S100 proteins coordinate a broad range of physiological processes, with particularly prominent roles in regulating tumour proliferation and invasion [23]. As a member of this family, S100A11 has been reported to associate with adverse prognosis across multiple cancer types [7, 24, 25]. Nevertheless, the functional pathways through which S100A11 operates in CRC, and its precise clinical significance, have not been fully elucidated. In the present study, IHC quantification revealed a significant elevation of S100A11 protein in advanced CRC specimens (Grade 3/Stage IV), which correlated closely with shortened overall survival. Of note, S100A11 upregulation was independent of KRAS and NRAS mutation status, and its high expression was preferentially associated with the MSS/MMR-proficient molecular context, pointing to its potential utility as a prognostic biomarker and therapeutic target in the RAS-mutant and MSS-dominant CRC populations that are largely unresponsive to current targeted and immune therapies. Taken together, the available evidence suggests that S100A11 functions as an oncogene in CRC, although the underlying molecular mechanisms warranted further systematic investigation.

Aberrant activation of the PI3K/AKT/NF-κB pathway is closely associated with tumour progression and therapeutic resistance across multiple solid tumour types [19], yet the upstream protein-level regulatory mechanisms governing this dysregulation in CRC remain incompletely defined. In hepatocellular carcinoma, S100A11 has been reported to promote metastasis through AKT and NF-κB activation [26], suggesting that the capacity of S100A11 to engage this signalling cascade is conserved across cancer lineages. The present study identifies the molecular basis for this activation in CRC: in contrast to mutation-driven direct PI3K activation (as seen with PIK3CA mutations), S100A11 mediates STRAP–PI3K complex formation through protein–protein interactions, representing a pathway activation mode that is dependent on protein complex assembly rather than on direct enzymatic gain-of-function.

In the present study, we identified STRAP as a binding partner of S100A11 in CRC. STRAP is overexpressed in several cancer types and was originally characterised as a TGF-β receptor-associated protein that contributes to neoplastic transformation by antagonising the antiproliferative arm of TGF-β signalling [20, 27]. Prior understanding of STRAP function has been largely confined to its role in TGF-β/Smad regulation, while its functional contribution to the PI3K/AKT pathway has remained unclear. It is noteworthy that within the non-Smad branch of TGF-β signalling, PI3K/AKT activation has been shown to be critical for TGF-β-induced EMT [28]; however, whether STRAP directly participates in PI3K activation had not previously been reported. The present study provides functional evidence for a TGF-β-independent role of STRAP in the PI3K/AKT pathway: through complex formation with S100A11, STRAP directly recruits PI3K to activate downstream AKT/NF-κB signalling. This finding broadens the oncogenic repertoire of STRAP and challenges its conventional perception as a TGF-β co-regulator. The strong co-expression and co-localisation of S100A11 and STRAP in the clinical cohort, together with their shared association with adverse prognosis, further supports a cooperative role for the two proteins in CRC malignant transformation. Regarding the regulation of STRAP protein stability, this study identifies K246 as the critical ubiquitination site at which OTUB1 protects STRAP from proteasomal degradation. The non-canonical mechanism by which OTUB1 suppresses E2 ubiquitin-conjugating enzyme activity through a non-catalytic mode (rather than direct ubiquitin chain cleavage) has been established for several oncogenic substrates [16]; the S100A11–OTUB1–STRAP regulatory axis described here provides a new instance of this mechanism operating in CRC. Notably, STRAP K246 lies within the WD6-7 domain (residues 221–302), which broadly overlaps with the S100A11-binding interface, raising the possibility that S100A11 sterically hinders ubiquitin ligase recognition of K246. This spatial protection model bears conceptual resemblance to chaperone-mediated substrate deubiquitination, whereby occupation of a protein interaction interface physically restricts ubiquitin ligase access—a principle documented in the CHIP/HSP90 protection system [29]—although direct biochemical evidence that S100A11 performs an analogous function awaits confirmation through in vitro reconstitution experiments.

NF-κB p65 serves as a master transcription factor governing proliferation, EMT, and inflammation-related gene programmes in CRC, and its sustained activation in multiple epithelial malignancies is a well-established correlate of poor prognosis [30, 31]. The ChIP-qPCR and dual-luciferase reporter assays presented here directly demonstrate that p65 transcriptionally activates both the S100A11 and STRAP promoters, and the feedforward loop thereby established converts a single upstream activation event into a continuously amplified output. Whereas S100A11 upregulation in gastric cancer has been attributed to HGF-driven induction [32], a recent study confirmed that NF-κB directly binds the S100A11 promoter to regulate its transcription, and that targeting the S100A11/NF-κB axis suppresses colon cancer progression [33]. Consistent with that report, the present data demonstrate that S100A11 transcription in CRC is directly controlled by NF-κB p65 and that its pro-tumourigenic effects depend on STRAP-mediated PI3K activation rather than HGF/MET signalling. This tissue-specific regulatory divergence suggests that the upstream control of S100A11 is cancer-type dependent, and the degree to which NF-κB p65-driven S100A11 transcription predominates in CRC warrants systematic evaluation in larger independent cohorts. It is also worth considering that the S100A11/STRAP/NF-κB feedforward circuit identified here may function as a dual-output regulatory hub: while mediating cell-autonomous oncogenic signalling through PI3K/AKT activation, it may simultaneously sustain chronic inflammation in the tumour microenvironment via NF-κB-dependent cytokine secretion (e.g., IL-6, TNF-α) [34]. Recent work has further reported that the S100A11–RAGE axis promotes immune evasion in colon cancer by recruiting immunosuppressive cells [35]. However, the specific contribution of S100A11 to immune microenvironment remodelling was not directly interrogated in the present study, and these inferences require evaluation in immunocompetent syngeneic tumour models. Compared with previously described NF-κB self-amplification loops [34], the cross-protein cascade feedback uncovered here (S100A11 → STRAP → PI3K → NF-κB → S100A11) may represent a more complex regulatory hierarchy and offers a distinct entry point for overcoming therapeutic resistance. We directly tested the functional necessity of this loop through genetic rescue experiments in a p65-knockdown background. Overexpression of either S100A11 or STRAP partially restored the proliferative and EMT deficits caused by p65 loss, yet neither fully returned cells to control-level activity. This incomplete rescue implies that the integrity of the entire loop, rather than activation at any single node, is required to sustain continuous pathway output. This behaviour is consistent with the documented ability of NF-κB to maintain persistent activation through multi-node redundancy in inflammation and cancer [36], and it further suggests that targeting any single component of this circuit in isolation may be insufficient to overcome resistance—pointing to the potential clinical value of combinatorial blockade strategies. A question worth addressing is whether the oncogenic phenotypes attributed here to the PI3K/AKT/NF-κB axis might also reflect contributions from parallel signalling branches. The present study does not exclude crosstalk with RAS/MAPK or Wnt/β-catenin pathways, both of which are frequently dysregulated in CRC and share transcriptional targets with NF-κB. Notably, the LY294002 rescue experiments demonstrate that PI3K inhibition only partially reverses S100A11-driven phenotypes, a result consistent with residual signalling through alternative routes. Systematic pathway profiling—such as phosphoproteomic screening in S100A11-perturbed cells across multiple CRC molecular subtypes—would be needed to quantify the relative contribution of each branch and to determine whether PI3K/AKT/NF-κB dominance is subtype-dependent.

Through multi-omics integration and functional validation, the present study establishes the S100A11/STRAP axis as a core regulatory node in CRC. Several limitations of the current work merit objective consideration. Although the series of genetic rescue experiments provides reasonably complete functional and causal evidence for the S100A11/STRAP/PI3K/AKT/NF-κB cascade, mass spectrometry-based direct identification of STRAP K246 ubiquitination and in vitro deubiquitination assays using purified proteins have not yet been completed, and atomic-resolution structural information on the ternary complex is similarly lacking; these higher-order biochemical and structural validations remain priorities for future work. Furthermore, the xenograft models employed here rely on immunodeficient hosts and therefore cannot recapitulate the full tumour immune microenvironment; the potential contribution of the S100A11–RAGE axis to immune evasion through recruitment of suppressive immune cells [35] was not assessed in this study, and further validation in syngeneic models or patient-derived organoids will be necessary for a comprehensive understanding of the in vivo significance of these mechanisms. At the clinical level, two interconnected gaps constrain the translational reach of the present findings. First, the scRNA-seq-derived identification of S100A11 as a malignant epithelial driver rests on public datasets; confirmation in an independent prospective cohort with fresh or frozen tissue and complete clinicopathological annotation would strengthen confidence in its generalisability. Second, the IHC analyses presented here capture individual marker distributions but do not permit formal mediation modelling: simultaneous quantification of S100A11, STRAP, p-AKT, and nuclear p65 on the same tissue microarray sections, followed by multivariable Cox regression and mediation analysis, would be needed to establish whether STRAP and downstream pathway nodes statistically mediate the relationship between S100A11 expression and patient survival. Addressing both gaps—through expanded prospective cohorts and multiplex quantitative IHC platforms—remains a priority for future work. Resolving these outstanding questions will further refine our understanding of the S100A11 regulatory network in CRC and lay the groundwork for the rational design of precision therapeutic strategies.

## Conclusion

In summary, this study systematically defines the molecular network through which S100A11 drives CRC progression by stabilising STRAP, activating the PI3K/AKT/NF-κB signalling pathway, and sustaining its amplification through a self-reinforcing feedforward loop. The marked overexpression of S100A11 in CRC tissue and its significant association with poor patient outcomes point to its potential clinical utility as a prognostic biomarker. The S100A11/STRAP binding interface and the STRAP K246 ubiquitination site represent candidate therapeutic targets, and combinatorial strategies directed at this axis alongside existing treatment modalities warrant further exploration in patient-derived organoid models (Fig. 6P).

## Materials and methods

### Cell culture

All cell lines were obtained from Procell Life Science & Technology Co., Ltd. (Wuhan, China) and authenticated by short tandem repeat (STR) profiling and mycoplasma contamination detection. NCM460, HCT15, SW620, SW480, HCT15, and 293 T cell lines were routinely cultured in high-glucose Dulbecco’s Modified Eagle Medium (DMEM, MA0212, Meilunbio, China). The Lovo cell line was maintained in the F12K medium (MA0230, Meilunbio, China), and HCT116 cells were also cultured in PRMI 1640 medium (MA0215, Meilunbio, China). All media were supplemented with 10% foetal bovine serum (FBS; PWL001, Meilunbio, China) and 1% penicillin–streptomycin (MA0342, Meilunbio, China), and cells were incubated at 37 °C under 5% CO₂.

### Cell transfection

Target plasmids or siRNAs were transiently delivered into CRC or HEK293T cells at 70–80% confluency using Lipo8000 (C0533, Beyotime, China) per the manufacturer’s protocol, and cells were harvested 48 h later for WB or qPCR. For stable line generation, lentiviral particles were co-transfected with packaging plasmids using PEI. Fresh medium containing puromycin (2 μg/mL) was applied 48 h post-transfection, and selection was maintained for 7 days. Stable monoclonal colonies confirmed by WB were expanded for subsequent experiments.

### CCK-8 assay

CRC cells were seeded in 96-well plates at 1 × 10³ cells/well (100 μL) and cultured under standard conditions (37 °C, 5% CO₂). At designated time points, 10 μL CCK-8 reagent (HY-K0301, MCE, USA) was added per well, followed by 2-h incubation. Absorbance at 450 nm was measured to assess viability.

### Colony formation assay

Stably transfected CRC cells (1 × 10³/well) were plated in 6-well plates. Medium was refreshed every 2–3 days. After 14 days, colonies were fixed with 4% PFA and stained with crystal violet. Colonies were photographed and quantified using ImageJ.

### Wound healing assay

Confluent CRC cells in 6-well plates were scratched with a sterile 10 μL tip. After PBS washing to remove debris, wound closure was monitored at indicated intervals and quantified using ImageJ.

### Transwell assay

Cell migration and invasion were assessed using Transwell chambers (8 μm, Corning, USA). For migration assays, 1 × 10⁵ CRC cells in 200 μL serum-free medium were seeded in the upper chamber, with 500 μL 10% FBS medium in the lower chamber. After 24 h, cells were fixed with 4% PFA (20 min), non-migrated cells were removed by cotton swab, and membranes were stained with 0.1% crystal violet (20 min). For invasion assays, Matrigel (082704, ABW Bio, China) diluted 1:8 was added to the upper chamber, and the rest was the same as the migration assay. Three random fields were selected under an inverted microscope for cell counting and analysis.

### Flow cytometry analysis

Logarithmically growing CRC cells from different treatment groups were prepared into single-cell suspensions, stained with the Annexin V-APC/PI apoptosis kit (AP107, Liankebio, China) according to the manufacturer’s instructions, and then analyzed by flow cytometry.

### Immunofluorescence

CRC cells were seeded in confocal dishes, fixed with 4% PFA at room temperature (RT) for 15 min, permeabilized with 0.1% Triton X-100 (P0096, Beyotime, China) for 10 min, and blocked with 5% skim milk at RT for 1 h. Primary antibodies S100A11 (AB180593, Abcam, USA) and STRAP (627712-1-Ig, Proteintech, China) were applied overnight at 4°C. After PBS washes, cells were incubated with Alexa Fluor-conjugated secondary antibodies at RT in the dark for 1 h, counterstained with DAPI for 10 min (C1002, Beyotime, China), and imaged using a confocal microscope (Olympus, Japan).

### Real-time quantitative PCR (RT-qPCR)

Total RNA was extracted from CRC cells using Trizol reagent (R401-01, Vazyme, China) according to the manufacturer’s instructions. cDNA was synthesized using the Hifair® Ⅲ 1st Strand cDNA Synthesis SuperMix for qPCR kit (11141ES60, YeSEN, China) with mRNA as the template. RT-qPCR analysis was performed using the Hieff® qPCR SYBR Green Master Mix to prepare the SYBR qPCR premix (11202ES08, YeSEN, China). The primer sequences used in this study are provided in the Supplementary Table 2.

### Cytoplasmic and nuclear protein isolation

Nuclear and cytoplasmic proteins were isolated using the Nuclear and Cytoplasmic Protein Extraction Kit (P0027, Beyotime, China) according to the manufacturer’s instructions.

### Western blotting (WB)

Proteins were extracted from cells or tissues using RIPA lysis buffer, quantified via BCA assay, and denatured in 5× loading buffer at 100°C for 10 min. Samples were resolved by SDS-PAGE and transferred to PVDF membranes. After blocking with 5% skim milk, membranes were incubated overnight at 4°C with primary antibodies, followed by HRP-conjugated secondary antibodies. Protein signals were detected using ECL substrate and analyzed with ImageJ software. The antibody information used in this study is provided in the Supplementary Table 3. Full and uncropped western blots uploaded as Supplemental Material.

### RNA-Seq analysis

Total RNA was extracted from S100A11- overexpressing and control HCT116 cells, and mRNA libraries were constructed. Paired-end sequencing (2×150 bp) was performed on the Illumina NovaSeq X Plus platform. The reads were aligned to the reference genome using STAR software, gene expression levels were counted by HTSeq, and differentially expressed genes were screened by DESeq2 (|log2FC | >1, FDR < 0.05). Gene Set Enrichment Analysis (GSEA) and Kyoto Encyclopaedia of Genes and Genomes (KEGG) pathway analysis were performed using the ClusterProfiler package. The reference gene sets included C5 GO gene sets, C7 Hallmark gene sets, and the KEGG database. The GSEA enrichment results were visualized using the GseaVis R package, and the KEGG enrichment results were visualized using the ggplot2 package.

### Co-immunoprecipitation (CO-IP) and mass spectrometry analysis

Lovo cells transfected with Flag-S100A11 for 48 h were lysed. Co-immunoprecipitation experiments were carried out using the Pierce™ Co-Immunoprecipitation Kit (26149, Thermo Scientific, USA) according to the manufacturer’s instructions. The obtained protein solution was used for mass spectrometry analysis of interacting proteins. The IP protein solution was mixed with SDS-PAGE loading buffer and heated at 100 °C for 5 min for boiling denaturation. The interaction relationship between proteins was confirmed by WB.

### Cycloheximide (CHX) chase assay

Cells were treated with 100 μg/ml CHX (HY-12320, MCE, USA) for 0, 2, 4, and 8 h, respectively. Then, the cells were collected for protein extraction and Western blot analysis.

### Treatment with MG132 and chloroquine

To distinguish proteasomal from lysosomal degradation, stably transfected CRC cells were treated individually with the proteasome inhibitor MG132 (10 μM; HY-13259C, MCE, USA) or the lysosomal inhibitor chloroquine (CQ; 20 mM; HY-17589A, MCE, USA) for 24 h, followed by protein extraction and WB analysis.

### Ubiquitination analysis

Lovo cells were co-transfected with a His-tagged ubiquitin expression vector (Taiting, Shanghai, China) and the recombinant plasmid of the target gene, and cultured continuously for 48 h. To inhibit protein degradation, 10 μM of the proteasome inhibitor MG132 was added to the transfected cells 6 h before collection. After cell lysis, immunoprecipitation was performed with appropriate antibodies, and the ubiquitination level was evaluated by Western blot detection.

### Surface plasmon resonance (SPR)

SPR experiments were performed on a Biacore 1 K instrument (Cytiva). STRAP protein was immobilised on a CM5 sensor chip via standard amine coupling: flow cell 2 was activated by injection of EDC/NHS mixture at 10 μL/min, followed by injection of STRAP protein diluted to 50 μg/mL in sodium acetate buffer (pH 4.5); residual active groups were then blocked with ethanolamine. Flow cell 1 served as the reference channel and was processed identically using protein-free acetate buffer in place of the protein solution. All experiments were conducted in 1× PBS-P+ (pH 7.4) supplemented with 5% (v/v) DMSO as the running buffer. Analytes (S100A11 and PI3K) were prepared as twofold serial dilutions ranging from 0.3125 to 10 μM in 96-well plates and injected sequentially from lowest to highest concentration at a flow rate of 30 μL/min, with an association phase of 60 s and a dissociation phase of 90 s. The chip surface was regenerated between each concentration cycle with 10 mM glycine-HCl (pH 2.0). Solvent correction was applied using a DMSO calibration curve spanning 4.5–5.8%. Sensorgrams were double-referenced against the reference flow cell, and global fitting using a 1:1 Langmuir binding model was performed in Biacore Insight Evaluation Software (Cytiva) to extract the association rate constant (ka*ka*), dissociation rate constant (kd*kd*), and equilibrium dissociation constant (KD=kd/ka*KD*=*kd*/*ka*).

### Chromatin immunoprecipitation (ChIP) assay

Cells were cross-linked with 37% formaldehyde at RT for 10 min, and the reaction was terminated by incubating with 10x glycine for 5 min. The cells were then washed with a mixture of pre-cooled PBS and protease inhibitors (C600387-0001, Sangon Biotech, China). The cells were collected using a cell scraper, centrifuged at 1500 rpm at 4 °C for 5 min, and the supernatant was discarded. Subsequent purification of nuclear lysates and immunoprecipitation was performed using the SimpleChIP® Enzymatic Chromatin IP Kit (9005S, CST, USA) according to the manufacturer’s instructions. The final eluted DNA was analyzed by real-time fluorescence quantitative PCR using the PerfectStart Green qPCR SuperMix ( + DyeⅡ) kit (AQ601-24, TransGen Biotech, China) to determine the enrichment of target sites, with the input sample used as a normalization control. The primer sequences are as follows: S100A11 (forward: 5′-aatgcccagaatgagctcatac-3′, reverse: 5′-ctctccagcgatttaagcagtc-3′), STRAP (forward: 5′-GAAGAGGCAGTAGCAATCATCTG-3′, reverse: 5′-CTGAGACACTAGAAATAGAAGGGAC - 3′).

### Dual-luciferase reporter assay

Wild-type (WT) and mutant (Mut) human S100A11 and STRAP promoters were synthesized by Tsingke Biological Technology Co., Ltd. (Beijing, China) and cloned into the pGL3-Basic vector. The WT or Mut S100A11 or STRAP promoter plasmid and the control pRL-TK plasmid were co-transfected into HEK293T cells. The analysis was performed using the Dual-Luciferase Reporter Assay Kit (RG088S, Beyotime, China), and the activities of firefly luciferase (experimental reporter signal) and Renilla luciferase (internal reference signal) were detected and evaluated by a microplate reader.

### Animal studies

All animals were purchased from the Laboratory Animal Centre of Model Organisms, Nanjing Biocytogen Co., Ltd. (China). Animal studies were conducted by the protocol approved by the Ethics Committee of Qingdao Central Hospital (Ethics No. 2024-1087).

The sample size was estimated based on preliminary experimental results. Prior to cell injection, animals were allocated to experimental groups using a simple randomization method. For subcutaneous xenograft models, 5×10⁶ stably transfected CRC cells in 100 μL PBS were injected into the right flank of 5-week-old female BALB/c-nu mice. Tumour volume was monitored twice weekly using calipers and calculated as Volume=1/2 (Length × Width²). Mice were sacrificed at endpoint for tumour collection and analysis. Tumour volume measurement and tumour weighing were performed by investigators blinded to group assignment (blinded assessment). For lung metastasis assays, 1×10⁶ cells were injected into the tail vein of NCG mice. After 28 days, lungs were dissected, and metastatic nodules were quantified following H&E staining. Lung metastatic nodules were counted under blinded conditions, with the counter unaware of sample group information.

### CRC tissue microarray

CRC tissue microarrays (HColA180Su23 and HColA160Su02) were purchased from Shanghai Outdo Biotech Co., Ltd. The HColA180Su23 array contains 75 paired samples of colorectal cancer tissue and adjacent normal tissue, while the HColA160Su02 array contains 100 such paired samples. All samples were collected with the informed consent of the patients and approved by the Biomedical Ethics Review Committee of Shanghai Outdo Biotech Co., Ltd. (Approval Nos.: SHYJS-CP- 230903; SHYJS-CP-1704001).

### Immunohistochemistry (IHC) and multiplex fluorescence

Sections underwent antigen retrieval in citrate buffer (pH 6.0), blocked with 3% H₂O₂ and 5% BSA, then incubated with anti-S100A11 overnight at 4 °C. HRP-conjugated secondary antibodies were applied for 45 min at RT, followed by DAB development and hematoxylin counterstaining. Staining intensity (0–3) and positive cell percentage (0-100%) were scored blindly, with a cutoff of 2.25 defining high- and low-expression subgroups. IHC staining of paraffin-embedded xenograft tissues followed the previously described protocol, with primary antibody incubation performed overnight at 4 °C.

For multiplex staining, Opal 7-PLEX kits were used with microwave-based antigen retrieval (AR6 buffer). Primary antibodies (S100A11 1:2,000, CK 1:10, STRAP 1:1,500) and Opal fluorophores were applied cyclically. DAPI-counterstained sections were imaged on a TissueFAXS SpectraS system for quantitative analysis.

### Bioinformatics analysis

Single-cell transcriptome data of normal colorectal tissues, adenomas, and cancer tissues were obtained from the Gene Expression Omnibus (GEO) database (GSE161277). Quality control, normalization, and UMAP dimensionality reduction clustering were performed using the Seurat package to identify cell subsets. Benign and malignant epithelial cells were distinguished by integrating inferCNV analysis. Cell developmental trajectory analysis was conducted using Monocle2 to screen pseudotime dynamic differentially expressed genes. For public CRC single-cell transcriptome datasets (GSE179784 and E-MTAB8107), subcellular localization analysis of S100A11 expression was performed via the TISCH online platform. Differential expression analysis of public CRC transcriptome datasets (TCGA, GSE37182, and GSE20970) was implemented using DESeq2. Full-length amino acid sequences of S100A11 and STRAP were retrieved from the UniProt database, with three-dimensional structures predicted by AlphaFold 3 and rendered using PyMOL.

### Statistical analysis

For normally distributed variables, group comparisons used independent samples t-tests (two groups) or one-way ANOVA (multiple groups). Non-normally distributed data were analyzed using Mann-Whitney U tests (two groups) or Kruskal-Wallis tests (multiple groups). Pearson linear regression evaluated variable correlations. Survival curves were constructed via the Kaplan-Meier method, with group differences determined by log-rank tests. Data are expressed as mean ± SEM. Statistical calculations and visualizations utilized GraphPad Prism (Version 9.4.1). The significance thresholds are set as *P < 0.05, **P < 0.01, ***P < 0.001, ****P < 0.0001, and “ns” (*P* ≥ 0.05) for values that do not reach significance.

## Supplementary information

Table S1

supplmentary material

## Data Availability

All data supporting the conclusions of this study are presented within the main text and/or supplementary materials. The datasets and materials used in this research are available from the corresponding author upon reasonable request.
