# Supplementary material for: S100A11 sustains STRAP stability to drive PI3K/AKT/NF-κB feedforward signalling circuit activation in colorectal cancer progression
Source: Cell Death Dis. 2026 Aug 12;17(1):952. doi: 10.1038/s41419-026-09117-x (PMC13748668; doi:10.1038/s41419-026-09117-x)
Supplement: Supplementary file 1 [file 41419_2026_9117_MOESM1_ESM.docx]

Supplementary Materials for

S100A11 sustains STRAP stability to drive PI3K/AKT/NF-κB feedforward signaling circuit activation in colorectal cancer progression

Jie Li ^1^^†^, Han Yang ^2, 3†^, Ziyuan Xu ^1†^, Hao Dong ^1^, Zhaojun Kou ^1^, Linlin Lu ^4,^ *, Xuezhen Ma ^1,^ *

Correspondence to: Lulinlin2007@hotmail.com

maxuezhen@qdu.edu.cn

**This PDF file includes:**

Figures. S1 to S6

Tables S1 to S3

Figure. S1. Single-cell transcriptomic characterization of colorectal tissues. (A) UMAP visualization of 13 cellular subtypes identified in normal colon, adenoma, and carcinoma tissues. (B) Tissue origin mapping of cell clusters. (C) Feature gene expression patterns across clusters. (D) Dot plot displaying marker gene signatures for major subpopulations. (E) Proportional distribution of cellular subtypes among histopathological groups. (F) InferCNV analysis delineating chromosomal copy number variations (CNVs) in epithelial cells, with immune cells as controls. (G) UMAP projection integrating epithelial subclusters, tissue origins, and CNV scores. (H) Heatmap of pathway enrichment across epithelial subpopulations. (I) Pseudotemporal trajectory analysis of C1, C4, and C6 subclusters.

Figure. S2. Identification of key genes driving colorectal carcinogenesis. (A) Heatmap of differentially expressed genes arranged by pseudotime and results of enrichment analysis. (B) Expression dynamics of the top six candidate genes for the fate of cancerous cells along the pseudotime trajectory.

Figure. S3. S100A11 expression correlates with mutational and microsatellite instability status in CRC. (A) Comparison of S100A11 mRNA expression between KRAS-mutant and KRAS-wild-type tumors in the TCGA-COADREAD cohort. (B) Spearman correlation analysis between S100A11 and KRAS mRNA expression. (C) Spearman correlation analysis between S100A11 and NRAS mRNA expression. (D) Comparison of S100A11 mRNA expression between NRAS-mutant and NRAS-wild-type tumors in the TCGA-COADREAD cohort. (E) Spearman correlation analysis between S100A11 and BRAF mRNA expression. (F) Comparison of S100A11 mRNA expression between BRAF-mutant and BRAF-wild-type tumors in the TCGA-COADREAD cohort. (G) Spearman correlation analysis between S100A11 mRNA expression and MSIsensor score. (H) Heatmap illustrating the correlations between S100A11 and the expression of MMR proteins MLH1, MSH2, MSH6, and PMS2. **P* < 0.05, ***P* < 0.01, ****P* < 0.001.

Figure. S4. Functional validation of S100A11 oncogenicity. (A) GSEA enrichment analysis. (B) RT-qPCR confirmation of S100A11 mRNA levels. (C) RT-qPCR analysis of EMT and apoptosis markers (E-cadherin, N-cadherin, Vimentin, Bcl-2). (D) Subcutaneous xenograft images and tumor metrics in OE-Control versus OE-S100A11 HCT116 groups. (E) Lung metastasis specimens, HE-stained images, and statistics of the number of lung metastases in the OE con and OE S100A11 groups of mice.

Figure. S5. Clinical correlates of S100A11-STRAP axis. (A) Top 10 proteins from mass spectrometry analysis of S100A11-interacting proteins. （B）Co-IP and WB analysis revealed interactions between exogenous S100A11 and STRAP in HCT116 cells.(C) Correlation analysis of S100A11 and STRAP expression in CRC tissue microarrays. (D) STRAP expression proportions across different pathological grades and clinical stages in CRC. (E) Kaplan-Meier survival analysis of STRAP expression. (F) Expression proportions of S100A11 and STRAP (high/low) across different pathological grades and clinical stages in CRC. (G) Kaplan-Meier survival analysis for high/low expression groups of S100A11 and STRAP. (H) Kaplan-Meier survival analysis for different combinations of S100A11 (high/low) and STRAP (high/low) expression.（I）Schematic of STRAP truncation mutants (J) RT-qPCR detected mRNA expression of EMT markers (E-cadherin, N-cadherin, Vimentin) and apoptosis marker Bcl2 in HCT116 cells treated with control, S100A11/STRAP co-transfection, or S100A11/STRAP 221-302 domain mutant co-transfection.

Figure. S6. (A) CCK-8 assay measuring proliferation of S100A11-stable-knockdown Lovo cells rescued with WT-STRAP, STRAP Δ221–302, STRAP K246R, myr-AKT, or CA-p65. (B) Colony formation assay assessing proliferation of the indicated Lovo cell groups. (C) Transwell invasion assay assessing invasiveness of the indicated Lovo cell groups. (D) Western blotting of representative apoptosis, EMT, and pathway markers in the indicated Lovo cell groups. (E–F) Immunoblot analysis demonstrating that S100A11 suppresses K63-linked polyubiquitination of STRAP at K246 in S100A11-depleted Lovo cells. (G–H) Proliferation of p65-knockdown Lovo cells overexpressing S100A11 or STRAP, assessed by CCK-8 (G) and colony formation assay (H). (I) Transwell invasion assay of p65-knockdown Lovo cells overexpressing S100A11 or STRAP. (J) Western blotting of representative apoptosis, EMT, and pathway markers in the indicated groups. **P* < 0.05, ***P* < 0.01, ****P* < 0.001, *****P* < 0.0001.

Table S1. Correlation between S100A11 expression levels and clinicopathological characteristics of CRC patients.

|  | Variables | S100A11 expression | | Total | *p* value |
| --- | --- | --- | --- | --- | --- |
|  |  | low | high |  |  |
| Sex |  |  |  |  | 0.772 |
|  | Male | 23 | 27 | 50 |  |
|  | Female | 23 | 24 | 47 |  |
| Age |  |  |  |  | 0.46 |
|  | ≤65 | 26 | 25 | 51 |  |
|  | >65 | 20 | 26 | 46 |  |
| Tumor_size |  |  |  |  | 0.151 |
|  | ≤5cm | 20 | 29 | 49 |  |
|  | >5cm  Unknow | 24 | 19 | 43  5 |  |
| Location |  |  |  |  | 0.631 |
|  | Left | 24 | 25 | 49 |  |
|  | Right  Unknow | 18 | 23 | 41  7 |  |
| T |  |  |  |  | 0.046 |
|  | T1-2 | 9 | 2 | 11 |  |
|  | T3 | 24 | 29 | 53 |  |
|  | T4 | 13 | 20 | 33 |  |
| N |  |  |  |  | 0.022 |
|  | N0 | 35 | 28 | 63 |  |
|  | N1a | 0 | 8 | 8 |  |
|  | N1b | 9 | 8 | 17 |  |
|  | N2a | 1 | 3 | 4 |  |
|  | N2b | 1 | 4 | 5 |  |
| M |  |  |  |  | 0.423 |
|  | M0 | 45 | 47 | 92 |  |
|  | M1 | 1 | 4 | 5 |  |
| TNM |  |  |  |  | 0.011 |
|  | Ⅰ-Ⅱ | 35 | 26 | 61 |  |
|  | Ⅲ-Ⅳ | 11 | 25 | 36 |  |
| Grade |  |  |  |  | <0.001 |
|  | Ⅰ | 37 | 19 | 56 |  |
|  | Ⅱ | 7 | 22 | 29 |  |
|  | Ⅲ | 2 | 10 | 12 |  |

Table S2. The list of primers used for qRT-PCR.

| **Name** | | **Sequence (5'--3')** |
| --- | --- | --- |
| GAPDH | Forward | GGGGAGCCAAAAGGGTCATCATCT |
|  | Reverse | GACGCCTGCTTCACCACCTTCTTG |
| S100A11 | Forward | CTGAGCGGTGCATCGAGTC |
|  | Reverse | TGTGAAGGCAGCTAGTTCTGTA |
| STRAP | Forward | GCGACCCGTGGTTGATTTG |
|  | Reverse | TGGCGTAGCATAGGTTTACCAT |
| E-cadherin | Forward | GCCCTGCCAATCCCGATGAAA |
|  | Reverse | GGGGTCAGTATCAGCCGCT |
| N-cadherin | Forward | AGCCAACCTTAACTGAGGAGT |
|  | Reverse | GACGCCTGCTTCACCACCTTCTTG |
| Vimentin | Forward | GACGCCATCAACACCGAGTT |
|  | Reverse | CTTTGTCGTTGGTTAGCTGGT |
| Bcl-2 | Forward | GGTGGGGTCATGTGTGTGG |
|  | Reverse | CGGTTCAGGTACTCAGTCATCC |
| NF-κB(p65) | Forward | ATGTGGAGATCATTGAGCAGC |
|  | Reverse | CCTGGTCCTGTGTAGCCATT |

Table S3. The list of indicated antibodies.

| **Antibody** | **Catalog numbers** | **Manufacturer** |
| --- | --- | --- |
| GAPDH | E-AB-40337 | Elabscience |
| β-catenin | BM0627 | Boster |
| Histone | A12477-2 | Boster |
| E-cadherin | ab231303 | Abcam |
| N-cadherin | ab76011 | Abcam |
| Vimentin | ab92547 | Abcam |
| Bcl-2 | R23309 | Zenbio |
| Caspase3 | 19677-1-AP | Proteintech |
| Cleaved-caspase3 | 9664T | Cell Signaling Technology |
| PARP | 13371-1-AP | Proteintech |
| Cleaved-PARP | ab32064 | Abcam |
| S100A11 | ab180539 | Abcam |
| S100A11 | ab169530 | Abcam |
| STRAP | 18277-1-AP | Proteintech |
| Ki-67 | 261202 | Zenbio |
| PI3K | AF4001 | Affinity |
| p-PI3K | 4228T | Cell Signaling Technology |
| p-PI3K | AF3242 | Affinity |
| AKT | 4691T | Cell Signaling Technology |
| p-AKT | 4060T | Cell Signaling Technology |
| NF-κB(p65) | 8242S | Cell Signaling Technology |
| p-NF-κB(p65) | 310013 | Zenbio |
| OTUB1 | R25227 | Zenbio |
| Anti-Ubiquitin | 80992-1-RR | Proteintech |
| Anti-Flag | A5712 | Selleck |
| Anti-Myc | 2276T | Cell Signaling |
| Anti-HA | abs137963 | Absin |
