# Supplementary material for: S100A11 sustains STRAP stability to drive PI3K/AKT/NF-κB feedforward signalling circuit activation in colorectal cancer progression
Source: Cell Death Dis. 2026 Aug 12;17(1):952. doi: 10.1038/s41419-026-09117-x (PMC13748668; doi:10.1038/s41419-026-09117-x)
Supplement: Supplementary file 2 [file 41419_2026_9117_MOESM2_ESM.docx]

Supplementary Materials for

S100A11 sustains STRAP stability to drive PI3K/AKT/NF-κB feedforward signaling circuit activation in colorectal cancer progression

Jie Li ^1^^†^, Han Yang ^2†^, Ziyuan Xu ^1^, Hao Dong ^1^, Zhaojun Kou ^1^, Linlin Lu ^3,^ *, Xuezhen Ma ^1,^ *

Correspondence to: Lulinlin2007@hotmail.com

maxuezhen@qdu.edu.cn

**This PDF file includes:**

Western blot raw data 1-10

Western blot raw data 1. (A) Raw data western blot Fig 1G. (B) Raw data western blot Fig 2B. (C) Raw data western blot Fig 2H. (D) Raw data western blot Fig 2O.

Western blot raw data 2. (A-B) Raw data western blot Fig 3C. (C-D) Raw data western blot supplementary Fig 4B. (E) Raw data western blot Fig 3G. (F) Raw data western blot Fig 3H.

**Western blot raw data 3.** (A) Raw data western blot Fig 3Q**.**

**Western blot raw data 4.** (A-B) Raw data western blot Fig 4B. (C) Raw data western blot Fig 4C. (D) Raw data western blot Fig 4D. (E) Raw data western blot Fig 4E. (F-G) Raw data western blot Fig 4G. (H) Raw data western blot Fig 4H.

**Western blot raw data 5.** (A) Raw data western blot Fig 4I. (B) Raw data western blot Fig 4J. (C) Raw data western blot Fig 4k. (D) Raw data western blot Fig 4L. (E-F) Raw data western blot Fig 4M. (G) Raw data western blot Fig 4N.

.

**Western blot raw data 6.** (A-B) Raw data western blot Fig 5B. (C) Raw data western blot Fig 5C.

**Western blot raw data 7.** (A) Raw data western blot Fig 5J. (B) Raw data western blot Fig 5K.

**Western blot raw data 8.** (A-B) Raw data western blot Fig 6A.

**Western blot raw data 9.** (A) Raw data western blot Fig 6B. (B) Raw data western blot Fig 6E. (C) Raw data western blot Fig 6F. (D) Raw data western blot Fig 6G. (E) Raw data western blot Fig 6J.

**Western blot raw data 10.** (A) Raw data western blot supplementary Fig 5D. (B) Raw data western blot supplementary Fig 5E. (C) Raw data western blot supplementary Fig 5F. (D) Raw data western blot supplementary Fig 5J.
